# Age trajectories of disability in instrumental activities of daily living and disability-free life expectancy among middle-aged and older adults in Taiwan: an 11-year longitudinal study

**DOI:** 10.1186/s12877-020-01939-4

**Published:** 2020-12-09

**Authors:** Wen-Ling Liao, Yu-Hung Chang

**Affiliations:** 1 Department of Physical Medicine and Rehabilitation, Taichung Tzu Chi Hospital, Buddhist Tzu Chi Medical Foundation, No. 66, Sec. 1, Fengxing Rd., Tanzi Dist., Taichung City 427, Taiwan; 2 Department of Public Health, China Medical University, No. 100, Sec. 1, Jingmao Rd., Beitun Dist., Taichung City 406, Taiwan

**Keywords:** Disability, Age trajectory, IADL, Middle-aged and older adults, Exercise, Social participation

## Abstract

**Background:**

This study aims to identify the age trajectories of disability in instrumental activities of daily life (IADLs) over 11 years and their correlates, and to estimate disability-free life expectancy for identified trajectory groups in middle-aged and older adults.

**Methods:**

We included 3118 participants aged 50 and over without IADL limitations at baseline from the Taiwan Longitudinal Study in Aging, followed across 1996–2007. We used group-based trajectory models to identify age trajectories of IADL disability, and multiple logistic regressions to examine their correlates. Sullivan method was used to compute IADL disability-free life expectancy for trajectory groups at different ages.

**Results:**

We identified two trajectories groups: 67.7% of participants classified as the late-onset group and 32.3% as the early-onset group. Female (adjusted odds ratio [aOR]: 1.93, 95% confidence interval [95% CI]: 1.54, 2.41), not being employed (aOR: 1.30, 95% CI: 1,08, 1,56), poor/fair self-rated health (aOR: 1.31, 95% CI:1.09, 1.58), hypertension (aOR: 1.32, 95% CI: 1.07, 1.63), diabetes mellitus (aOR: 2.29, 95% CI: 1.72, 3.07), arthritis (aOR: 1.42, 95% CI: 1.11, 1.81), stroke (aOR: 2.21, 95% CI: 1.04, 4.70), and one-point increase in a 10-item depression scale (aOR: 1.04, 95% CI: 1.02, 1.06) were associated with early-onset of disability, whereas higher education (aOR: 0.59, 95% CI: 0.42, 0.81), regular exercise (aOR: 0.76, 95% CI: 0.62, 0.93), and participating voluntary or club activities (aOR: 0.78, 95% CI: 0.65, 0.93) related to the late-onset. IADL disability-free life expectancies at 65 years old in the late-onset group were 15.6 years for women and 14.4 for men, respectively, comprising 56.6 and 64.2% of their remaining life, whereas those of the early-onset group were 4.8 and 4.6 years for women and men respectively, comprising 22.5 and 27.2% of remaining life.

**Conclusions:**

Early-onset of IADLs disability may correlate to chronic conditions, and engagement in employment, exercise, and social participation were associated with a reduced risk of early disability in IADLs.

**Supplementary Information:**

The online version contains supplementary material available at 10.1186/s12877-020-01939-4.

## Background

Advances in health care have increased human life expectancy. Longer life span, however, does not ensure an extended period of healthy time and good quality of life. Disabilities become more severe with increasing age [1–3]. Due to the high prevalence of disabilities among older adults, accelerated aging countries will suffer from increasing social and economic burdens [4]. The older population aged 65 and above in Taiwan has risen from 7.1% in 1993 to 14.5% in 2018 and will exceed 20.7% in 2026 [5]. Older people need to retain their ability to live independently in the community because the shrinking workforce may reduce the society’s financial capacity and care provision for them.

The World Health Organization has defined disability as negative outcomes resulting from interactions between individuals and their surrounding environment [6]. Disability is an umbrella concept that brings together physical impairments, activity limitations and participation restrictions influenced by contextual factors at the personal and environmental levels. Among older people, difficulty in activities of daily living (ADLs) is one of the most commonly assessed aspects of disability, since activities is the element of participation that determine the extent of participation and the way of interaction between persons with limitations and their living environment. Basic ADLs (BADLs) are the most basic self-care functions essential for an independent life and are mainly activities carried out at home; in contrast, instrumental ADLs (IADLs) tend to have a greater cognitive component, involve more interaction with one’s environment, and emphasize community activities [7, 8]. Compared with BADLs, IADLs more involve complicated cognitive and physical skills. As functions decline, IADL impairments usually precede BADL deficits [9]. In this case, analyzing functional decline in IADLs can help predict subsequent disability in BADLs.

Previous studies have examined disability among older adults. The prevalence of ADL disability and IADL disability among Taiwanese aged 65 years or older was 12–14% and 45–50%, respectively [10]. However, functional decline and preceding factors are common in middle-aged [11]. Early onset of disability may confer premature mortality and longer lifetime living with disability and higher costs in health care [12]. However, a prolonged lifespan in the aging population may not necessarily extend the duration of disability [13], as long as modifiable risk factors that lead to disability are intervened [14]. Therefore, it is crucial to observe functional changes from independence to the onset of disability and identify correlates of the functional decline at a younger age in the clinical and policy context.

The current study had the following aims: (a) to use group-based modeling methods to explore the development process of IADL disability over time, and to determine different IADL disability trajectory groups among the representative population of Taiwan aged 50 and over; (b) to examine associations of protective or risk factors at baseline with identified trajectory groups; (c) to compare the number of years of IADL disability-free life relative to total life expectancy and the extent of disability expansion or compression among the identified trajectory groups.

## Methods

### Data sources

We used data from the Taiwan Longitudinal Study in Aging (TLSA), initiated in 1989, with a representative sample including 4049 participants aged ≥60 years living in the community or institutions [15]. Five follow-up surveys were conducted in 1993, 1996, 1999, 2003, and 2007. Another 2462 participants aged 50–66 years were introduced in 1996 to supplement the loss of samples from death or loss to follow-up between 1989 and 1996, and to create a representative middle-aged sample ≥ 50 years of age. Participants were interviewed at home or via telephone. Response rates for all survey waves were over 80%. This study focused on middle-aged and older adults and examined survey data from 1996 onward. To have a clear causal relationship between baseline risk (or protective) factors and the subsequent onset of IADL disability, we excluded participants who already had IADL disabilities (*n* = 1760) or were living in institutions (*n* = 13) at baseline; participants who had only completed one of the four survey waves (*n* = 240) were also excluded due to the inability to measure changes in their IADLs. Our baseline analytical sample included 3118 respondents aged ≥50 years living in the community. In the last wave (2007), 2301 were alive and interviewed, 178 (5.7%) were alive but lost to follow up, and 639 (20.49%) had died before the last wave. Weights were provided to combine the original and supplemental cohorts to ensure the total group of 5131 participants was representative of individuals aged ≥50 years in Taiwan.

### Assessment of disability

The IADL measurement in TLSA was adapted from the Lawton’s IADL scale [8]; the Chinese version of the scale was reported to have established validity and reliability [16]. Respondents of TLSA were asked the question: “If you were to carry out the following tasks by yourself, would you have any difficulty with these tasks?” The examined activities were shopping, managing finances, taking public transportation, doing heavy housework, doing light housework, and telephoning. In the original questions, the difficulty level for each IADL activity was assessed using a 4-point scale (0 = *no difficulty, 1 = a little difficulty, 2 = very difficulty,* 3 = *unable to carry out*). In this study, responses were dichotomized to *no difficulty* (0) and *some difficulty* (1). IADL disability was defined as experiencing some difficulty carrying out one of the examined IADL tasks. Cronbach’s alpha of the 6-task IADL used in this study ranged from .82 to .92, demonstrating good internal consistency.

### Assessment of covariates

To examine factors affecting IADL disability trajectories, we collected personal baseline information from the 1996 survey. Demographics and socioeconomic status included age, sex, education level, ethnicity, place of residence, marital status, employment, satisfaction of economic status and housing tenure. Health-related behaviors comprised smoking, drinking alcohol, betel nut chewing, leisure-time exercise, habitual eating of breakfast, and receiving of health checks in the previous three years. Health conditions and comorbidities included history of hospitalization during the past year, self-rated health, self-reported visual impairment and hearing loss, and 15 self-reported chronic diseases or conditions: hypertension, diabetes, heart disease, stroke, cancer, bronchitis, arthritis, gastrointestinal disorders, liver or gall bladder problems, cataract, glaucoma, kidney diseases, gout, spin spur, and hip or other area fractures. Depressive symptoms were examined using the 10-item Center for Epidemiologic Studies Depression Scale (CES-D) [17]. Cognitive functions of participants were assessed using the recall test of a 10-word list and the digits backward test [18, 19]. Social participation was defined as attending any volunteer or club activities, while social isolation was defined as living alone.

### Statistical analysis

To identify distinct IADL disability trajectory groups, we used a generalized group-based trajectory model (GBT) to estimate trajectories of IADL disabilities and the probability of nonrandom dropout [20, 21]. IADL disability trajectories were estimated using a binary logistic model with age as the independent variable; nonrandom dropout was defined as death, whose probability was modeled as a function of IADL disability status at the survey wave prior to death [21]. Following Nagin [20], we choose the best fitting model based on the following criteria: the model with the lowest Bayesian Information Criteria value; and the model with the value of average posterior probabilities for each trajectory group > 0.7.

We subsequently analyzed associations between participants’ baseline characteristics and the identified IADL disability trajectory groups. Candidate factors were chosen using the following steps: first, we examined the associations of baseline variables with the trajectory groups using Chi-squared tests for categorical variables and independent t-tests for continuous variables. Second, those factors significantly associated with the trajectory groups were used to establish multivariable logistic regressions where odds ratios with 95% confidence interval were estimated.

Finally, in order to further examine the difference in disability-free life expectancy between the identified IADL disability trajectory groups, we used Sullivan’s life table method [22], combining age-specific mortality rates with disability probability, to estimate the number of years of IADL disability-free life, IADL-disabled years, and the ratio of IADL disability-free years to total life expectancy. Age-specific disability probabilities were calculated using the results of the IADL disability trajectory analysis; accordingly, the identified trajectory groups were associated with different disability probabilities at specific ages. We used Gompertz models to estimate mortality rates [23]; the model was adjusted according to each trajectory group and sex. Total life expectancy can be divided into the number of IADL disability-free years, and years with IADL disability. For detailed computing methods, referred to Jagger et al. [24].

## Results

**Fig. 1:**
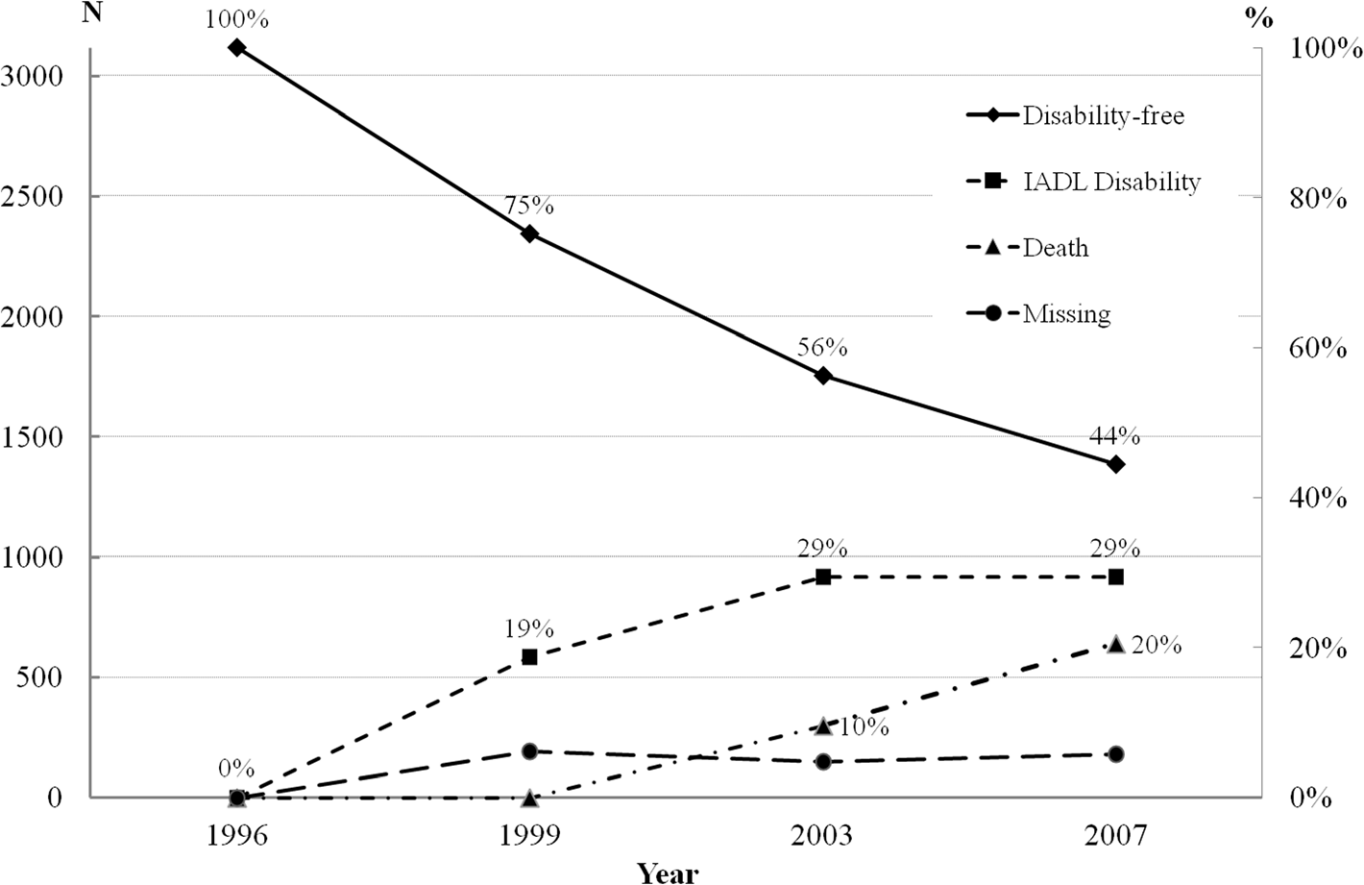
Numbers and percentages of IADL disability, disability-free and death in study participants, 1996–2007. N = 3118 at 1996; IADL: instrumental activity of daily life

At baseline, the mean age of 3118 participants (1901 male and 1217 female) was 60.7 (±7.9) years. Over half of participants were married (80.7%), having no junior high school education or above (71.8%), living in cities or suburbs (62.9%), not employed (52.5%), living in a house they owned (67.2%), unsatisfied with their economic status (61.0%), not living alone (93.3%) and without social participation (53.1%) (see Supplementary Table S1). About half of the participants’ self-assessed health was good (48.2%); in the past year, one in ten had a history of hospitalization. The most frequently reported health problems were hypertension (19.9%), followed by gastrointestinal ulcer (12.8%) and arthritis (12.6%); the rest of health problems were reported less than 10% among participants. Among the participants, one-third of were smokers, 27.5% had habitual alcohol drinking, and less than 10% had chewed betel nuts; more than 90% were used to eating breakfast, but did not get enough exercise (66.5%), i.e., three times a week and 30 minutes each time; 60% of them had not done a health check within three years. After the third year of follow-up, 19% of the participants had IADL disability; while in the seventh and eleventh years, the number of disabled people increased to 29% (Fig. 1).

**Fig. 2:**
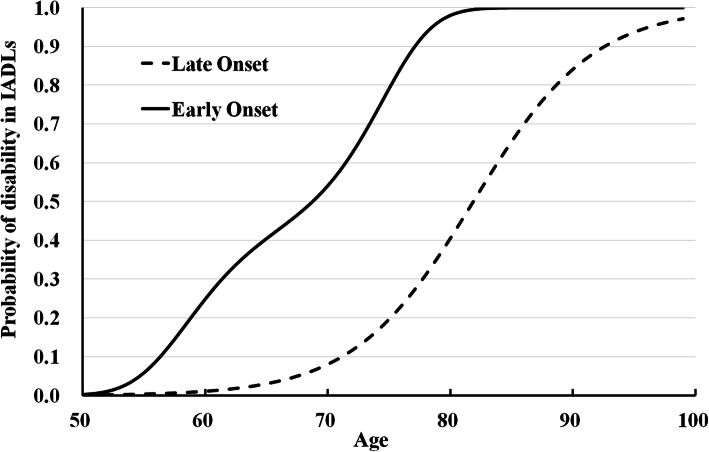
Age trajectories of the probabilities of IADL disability by the late-onset and early-onset groups. Age trajectories were estimated using generalized group-based trajectory models

Figure 2 illustrates the IADL disability trajectory groups representing the probability of IADL disability by age, estimated from the best-fitting model using the GBT method (Table 1). Two trajectory groups were identified: the late-onset group that was characterized by delayed IADL disability onset and the early-onset group characterized by early onset of disability. The late-onset trajectory includes an intercept and a linear scaled age parameter; in contrast, the early-onset group is a cubic polynomial of scaled age. The late-onset group’s size was greater than that of the early-onset group (67.7 and 32.3%, respectively). The probability of IADL disability increased continuously with age, independent of group membership. In the late-onset group, IADL disability became 25 and 50% likely at ages 77 and 82 years, respectively (a difference of 5 years). In contrast, in the early-onset group, these ages were 61 and 69 years, respectively (a difference of 8 years).

**Table 1:** Results of logistic regressions for age trajectories of IADL disability among study participants

	Group 1	Group 2
	Late onset	Early onset
Parameters for IADL disability trajectory		
Intercept	−2.140 (.127)^*^	0.399 (.138)^*^
Linear scaled age^§^	2.047 (.098)^*^	1.873 (.284)^*^
Quadratic scaled age^§^		1.685 (.492)^*^
Cubic scaled age^§^		1.050 (.240)^*^
Group size (%)	67.66 (2.867) ^*^	32.34 (2.867) ^*^
Average posterior probabilities	0.88	0.81
Bayesian Information Criterion = − 5132.56 (N = 3118)		

*Note.* Standard errors are in parentheses; ^*^p < .05; ^§^Scaled age = (age - 71.43) / 10; Group size represents the estimated proportion of the population corresponding to each group; average posterior probabilities for each trajectory group were > 0.7, indicating that the models were acceptable [20]

Table 2 presents the results of bivariate analysis and multivariate analysis. In bivariate analysis, participants in the early-onset group tended to be female, less educated, Hoklo, not employed, and unsatisfied with their economic status (*p* < 0.05). Regarding health status measures, participants in the early-onset group tended to have fair-to-poor self-rated health, be hospitalized in the previous year, score lower (worse) in cognitive tests, score higher in CES-D (more depressive), or have a chronic disease, but were less likely to smoke, drink alcohol, exercise regularly, and attend volunteer or club activities (*p* < 0.05). Non-significant factors included house ownership, living area, marital status, betel nut chewing and breakfast habits, hearing impairment, cancer, bronchitis, cataract, and glaucoma.

**Table 2:** Results of the bivariate and multivariate analysis to examine associations of baseline risk/preventive factors with trajectory groups among participants of the Taiwan Longitudinal Study in Aging (*N* = 3118)

	Bivariate Analysis^†^			Multivariate Analysis^a^		
	Trajectory Groups			Outcome: Early Onset		
**N**	Late onset 2110	Early onset 1008				
Variables	%	%	*P* value	OR	95% CI	*P* value
Female	35.0	57.5	<.0001	1.93	1.54–2.41	<.0001
Education level			<.0001			
Uneducated	22.8	36.7		Ref.		
Primary school	45.0	45.4		0.87	0.71–1.06	0.1646
Junior high school	12.3	9.2		0.76	0.56–1.05	0.0917
Above senior high school	19.9	8.8		0.59	0.42–0.81	0.0013
Ethnic groups			<.0001			
Hoklo	66.9	73.1		Ref.		
Hakka	18.0	15.5		0.89	0.70–1.12	0.3115
Mainlander	14.0	9.1		0.75	0.55–1.02	0.0665
Other	1.2	2.4		1.78	0.95–3.34	0.0713
Not employed	48.4	63.4	<.0001	1.30	1.08–1.56	0.0051
Unsatisfied with economic status	58.7	67.0	<.0001	1.07	0.89–1.29	0.4685
Poor/fair self-rated health	46.6	65.6	<.0001	1.31	1.09–1.58	0.0042
Smoking	33.5	24.8	<.0001	1.12	0.89–1.41	0.3259
Drinking alcohol	30.5	19.6	<.0001	0.84	0.67–1.05	0.1161
Hospitalization	9.1	13.9	<.0001	1.10	0.83–1.47	0.4965
Visual impairment	6.7	11.7	<.0001	1.28	0.98–1.66	0.0689
Hypertension	17.9	25.3	<.0001	1.32	1.07–1.63	0.0087
Diabetes	5.4	13.0	<.0001	2.29	1.72–3.07	<.0001
Heart disease	7.1	10.8	0.0003	1.05	0.78–1.42	0.7402
Stroke	0.8	1.8	0.0081	2.21	1.04–4.70	0.0396
Arthritis	10.2	18.9	<.0001	1.42	1.11–1.81	0.0046
Gastrointestinal disorders	11.8	15.6	0.0027	0.94	0.73–1.20	0.597
Liver or gall bladder problems	3.7	6.4	0.0008	1.43	0.97–2.11	0.0693
Kidney diseases	3.4	7.3	<.0001	1.26	0.86–1.84	0.2428
Gout	5.0	8.9	<.0001	1.42	1.02–1.98	0.0393
Spin spur	4.4	9.1	<.0001	1.57	1.12–2.20	0.0083
Hip fractures	0.5	1.8	0.0002	2.43	1.05–5.60	0.0374
Other area fractures	1.4	2.6	0.0144	1.24	0.69–2.25	0.4732
Regular exercise			<.0001			
None	45.3	53.1		Ref.		
≤2 times/week, < 30 mins/time	3.0	3.0		0.85	0.52–1.39	0.52
≤2 times/week, ≥30 mins/time	3.6	2.1		0.69	0.40–1.17	0.1652
≥3 times/week, < 30 mins/time	12.6	13.4		0.91	0.70–1.18	0.4786
≥3 times/week, ≥30 mins/time	35.4	28.3		0.76	0.62–0.93	0.0089
Recall test, mean(s.d)^§^	4.8(2.4)	4.4(2.4)	<.0001	0.87	0.71–1.08	0.2061
Backward digit test	31.8	23.1	<.0001	0.98	0.94–1.02	0.3092
CES-D Scores, mean(s.d)^§^	3.7(4.7)	5.7(5.9)	<.0001	1.04	1.02–1.06	<.0001
Social participation	49.6	39.8	<.0001	0.78	0.65–0.93	0.0048

Chi-squared test for categorical variables; independent t test for continuous variables

^a^Multiple logistic regressions; predicted outcome: early onset of IADL disability; *OR* odds ratio; *95% CI* 95% confidence interval

Note: all calculations were weighted to compose a representative sample of Taiwan’s population of individuals aged ≥50 years; only baseline factors significantly associated with group membership are presented

The multivariate logistic regression analysis included only factors significantly associated with disability group membership in the bivariate analysis. Compared with the late-onset group, early-onset group members were more likely to be female (adjusted odds ratio [aOR]: 1.93, 95% Confidence Interval [95% CI]: 1.54, 2.41), not employed (aOR:1.30, 95% CI: 1.08, 1.56), fair-to-poor self-rated health (aOR: 1.31, 95% CI: 1.09, 1.58), and tended to have hypertension (aOR: 1.32, 95% CI: 1.07, 1.63), diabetes (aOR: 2.29, 95% CI: 1.72, 3.07), strokes (aOR: 2.21, 95% CI: 1.04, 4.70), arthritis (aOR: 1.42, 95% CI: 1.11, 1.81), gout (aOR: 1.42, 95% CI: 1.02, 1.98), spin spur (aOR: 1.57, 95% CI: 1.12, 2.20), and hip fractures (aOR: 2.43, 95% CI: 1.05, 5.60), and higher CES-D scores (aOR of a one-point increase: 1.04, 95% CI: 1.02, 1.06). Participants were less likely to be in the early-onset group if they had education above senior high school (aOR: 0.59, 95% CI: 0.42, 0.81), exercise ≥3 times a week for over thirty minutes each time (aOR: 0.76, 95% CI: 0.62, 0.93), or attended volunteer or club activities (aOR: 0.78, 95% CI: 0.65, 0.93).

**Fig. 3:**
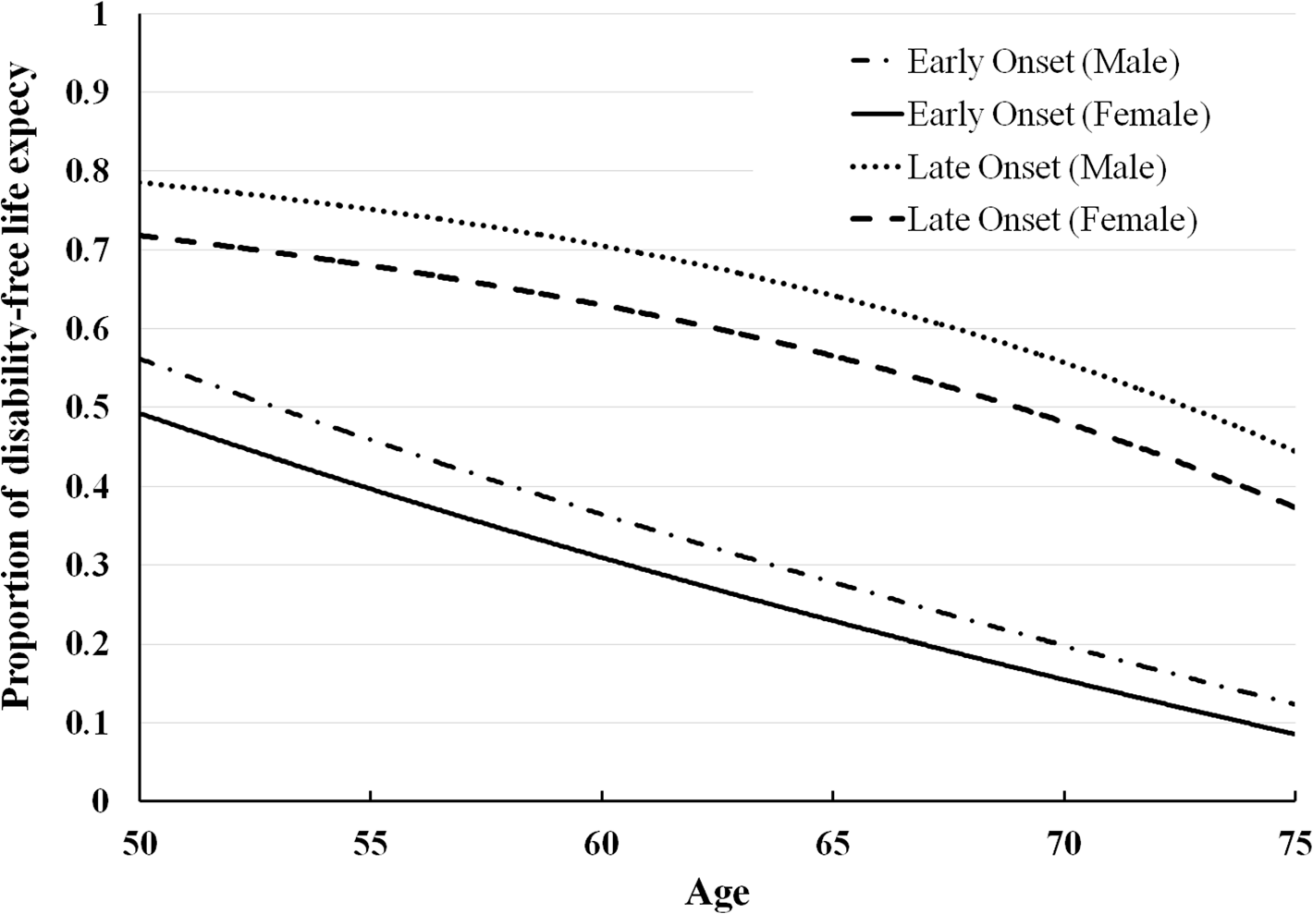
Proportions of disability-free life expectancy to life expectancy at several ages for men and women

We examined group membership’s effect on life expectancy by comparing the proportion of lifespan spent with IADL disability to total life expectancy at different ages for men and women (Table 3). At all ages, individuals in the late-onset group had a longer life expectancy as well as disability-free life expectancy than those in the early-onset group. Within each group, women had a higher life expectancy and disability-free life expectancy, but a lower ratio of disability-free life expectancy to life expectancy than men had. For example, women and men’s ratios of disability-free life expectancy to life expectancy at 65 years old were 0.57 and 0.64 in the late-onset group, respectively, and 0.22 and 0.27 in the early-onset group. We can observe this sex difference at different ages (Fig. 3).

**Table 3:** Estimates of IADL disability-free life expectancy and their proportions to life expectancy at different ages

	Men			Women		
Age	Life Expectancy^a^ (a)	Disability-free Life Expectancy (b)	(b)/(a) (%)	Life Expectancy (a)	Disability-free Life Expectancy (b)	(b)/(a) (%)
50 years old						
Late-onset group	35.7	28.1	78.6	41.4	29.7	71.8
Early-onset group	29.3	16.3	55.5	34.8	17.0	48.7
55 years old						
Late-onset group	31.1	23.4	75.1	36.6	24.9	68.0
Early-onset group	24.9	11.7	46.8	30.2	12.2	40.4
60 years old						
Late-onset group	26.6	18.8	70.5	32.0	20.2	63.0
Early-onset group	20.8	7.7	36.9	25.8	8.1	31.2
65 years old						
Late-onset group	22.4	14.4	64.2	27.5	15.6	56.6
Early-onset group	16.9	4.6	27.2	21.6	4.8	22.5

^a^Life expectancy at different ages was computed by life table where a mortality rate, M, at different ages, AGE, were estimated using Gompertz model: ln (*M*_*AGE,GROUP,SEX*_) = −11.247 + 0.099 x *AGE* + 0.689 x GROUP + (−0.592) x SEX, where GROUP denotes trajectory group memberships (0 = late onset, 1 = early onset), and SEX is sex (1 = female, 0 = male)

## Discussion

This study examined a representative sample of individuals aged ≥50 years in Taiwan to analyze trajectories of IADL disability from 1996 to 2007. This is the first study to analyze IADL disability trajectories from a point prior to disability incidence and using a model incorporating mortality. This study’s key findings are as follows.

We identified two distinct IADL disability trajectory groups (late-onset and early-onset) representing respectively 67.7 and 32.3% of the population. Previous studies based on different study populations have identified three or more trajectory groups [2, 25–30]. The difference in numbers of trajectory groups may stem from different study populations, measurements of disability, or modelling methods. Compared with these studies, the current study did not identify a persistent or continuing disability group because of the exclusion of subjects with baseline disability that prevented a reverse causality between baseline correlates and the onset of IADL disability. Also, this study shows that the probability of disability increases with age regardless of the early onset or late onset of disability. No group of older people could live independently with their physical functions being not affected by increasing age in the long term, different from previous research [2, 25, 29, 30], yet consistent with LG Martin, Z Zimmer and J Lee [27]. This may be because the follow-up time of this study was long; those without physical limitations at baseline may eventually develop functional decline in the late period of follow-up.

Comparing probabilities of disability of two trajectory groups at ages between 50 and 70 years old, physical functions of early-onset group members deteriorated faster than those of late-onset group members. In addition, it took about 19 years for the prevalence of IADL disability to rise from 0 to 50% among early-onset group members, but the same change took 32 years among late-onset group members. This remarkable difference in time-to-onset suggests that if the onset of disability could be intervened to delay, the prevalence of disability would be reduced among older adults.

Our trajectory analysis indicated that it is possible to delay the onset of IADL disability through interventions targeting factors associated with early-onset disability. The current study found that participants who were not employed, less educated, and experienced more depressive symptoms were more likely to have early-onset disabilities, whereas those who had sufficient exercise (three times or more per week for over thirty minutes each time) or attended club or volunteer activities were more likely to experience late-onset disabilities. The Report of the Second World Assembly on Aging [31] highlighted the importance of opportunities for employment in satisfying and productive work and having access to education and training courses that promote and protect working ability for older persons. Participating in paid or unpaid work can improve health in many dimensions, such as mortality, functional independence, and mental health [32–34], although there exist age and sex differences in the beneficial effect on physical functions [34]. Given an increasing older population, reemployment of older people may decrease the burden on society and delay the onset of disability, particularly as employment would increase an older person’s opportunity for social participation [35], in turn reducing depressive symptoms [36]. The present results imply that policies or interventions to improve employment and social participation or reduce depression may prevent early-onset of IADL disability.

Compared with those with a lower level of engagement, participants who engaged in physical activities more than three times per week for more than thirty minutes each time significantly protected against early-onset group membership. Our results are in concordance with several systematic review studies that confirmed the protective effect of exercise on functional decline or sarcopenia in later life [37–40]. This result also supports the exercise recommendation for older adults made by the American College of Sports Medicine and the American Heart Association (i.e., that older adults should perform moderate-intensity aerobic exercise for at least 30 min, five days a week) [41]. However, some uncertainty remained regarding the required beneficial dosage (i.e., exercise types, intensity, frequency, and duration). The present results may therefore be used as a reference for exercise among middle-aged and older adults; however, future research should aim to determine optimal exercise patterns for middle-aged and older adults.

In bivariate analysis, lower performance in cognitive function, including digit backward test or recall test, had a significant association with early onset of IADL. However, after adjusting for other covariates, their significance diminished. There may be confounding factors, such as education or stroke that are related to both cognitive function and physical function. In addition, people with depression may show cognitive impairment on cognitive tests [42], and thus cognitive tests might not be able to distinguish cognitive impairment from depression [43]. Therefore, the association between cognitive function and IADL impairment may be attenuated when CES-D scores were adjusted for in the multivariate analysis.

The disability-free life expectancy at 65 years old for women and men in the late-onset group constituted 57 and 64% of their predicted remaining lifespan, whereas those figures in the early-onset group constituted 22 and 27% of their predicted remaining lifespan (Table 3). Japanese study estimated 65-year-old women and men’s disability-free life expectancy at 14.6 and 12.8 years, respectively, constituting 70 and 74% of their remaining life [44]. In Italy, the proportion of IADL disability-free to life expectancy among men and women aged 65 years was 77 and 63% [45]. The present figures for Taiwan were less than those for Japan and Italy. However, all these three studies revealed that men had a larger proportion of disability-free life expectancy, although women than men had a longer total life expectancy. Furthermore, regarding the two disability trajectory groups identified in this study, the late-onset group had a longer total life expectancy and a longer disability-free life expectancy. Improving population health through reducing comorbidities and promoting engagement in physical, social and economic activities would introduce a health transition from early-onset to late-onset of disability that achieves compression of morbidity [46, 47].

This study has the following limitations. First, due to the nature of the questionnaire, some factors associated with disability (e.g., compressive cognitive tests, falling history, body mass index, etc.) were not investigated. Second, the analyzed data has a 3–4-year survey window, and some transitions may not have manifested at the right time to be captured in one of the four waves. Third, IADL disability was defined as experiencing difficulty with any IADL task; however, age and sex may differentially affect onset of disability associated with specific IADL tasks that influence dependence of older persons in a different manner. Finally, the exclusion of participants who were IADL disabled at baseline that undermines the sample representativeness; therefore, the proportions of trajectory groups did not represent their prevalence in the population.

## Conclusions

The present results indicate that IADL disability is not inevitable before very old ages. We identified several factors that may postpone IADL disability. Interventions that target individuals in middle age to compress the morbidity period and improve longevity are promising. This information from the study can aid in developing effective policies aimed to promote successful aging in the health transition of extended longevity.

## Supplementary Information

**Additional file 1 Supplementary Table S1** pdf, baseline characteristics of the participants of the Taiwan Longitudinal Study in Aging.

## Data Availability

The data that support the findings of this study are available from the Health and Welfare Data Science Center (HWDC), Ministry of Health and Welfare, Taiwan, but restrictions apply to the availability of these data, which were used under license for the current study, and so are not publicly available. However, data may be obtained with the permission of the HWDC, and data requests can be submitted to the HWDC as formal proposals (**https://dep.mohw.gov.tw/dos/np-2497-113.html**). Applicants must abide by the Personal Data Protection Act and the relevant regulations of the Ministry of Health and Welfare, Taiwan.

## Abbreviations

ADL: Activity of Daily Life
BADL: Basic Activity of Daily Life
IADL: Instrumental Activity of Daily Life
TLSA: The Taiwan Longitudinal Study in Aging
CES-D: The Center for Epidemiologic Studies Depression Scale
GBT: Group-Based Trajectory Model
