# Supplementary material for: Age trajectories of disability in instrumental activities of daily living and disability-free life expectancy among middle-aged and older adults in Taiwan: an 11-year longitudinal study
Source: BMC Geriatr. 2020 Dec 9;20:530. doi: 10.1186/s12877-020-01939-4 (PMC7727135; doi:10.1186/s12877-020-01939-4)
Supplement: Supplementary file 1 — Additional file 1 Supplementary Table S1 pdf, baseline characteristics of the participants of the Taiwan Longitudinal Study in Aging. [file 12877_2020_1939_MOESM1_ESM.pdf]

Supplementary Table S1. Baseline characteristics of the participants of the Taiwan Longitudinal Study in Aging (N=3,118)

Variables	N	*Unweighted* % *Mean ±(S.D.)*	*Weighted* % *Mean ±(S.D.)*
**Age**	3118	63.1(8.2)	60.7(7.9)
**Female**	1217	39.0	41.2
**Education level**			
**Not formally educated**	873	28.0	26.6
**Primary school**	1364	43.8	45.1
**Junior high school**	359	11.5	11.4
**Senior high school and above**	522	16.7	16.8
**Ethnic groups**			
**Hoklo**	2037	65.5	68.6
**Hakka**	531	17.1	17.3
**Mainlander**	500	16.1	12.6
**Other**	43	1.4	1.5
**Place of Residence**			
**Urban**	1221	39.6	39.7
**Suburban**	720	23.3	23.2
**Rural/remote area**	1145	37.1	37.1
**Married**	2434	78.1	80.7
**Not employed**	1813	58.2	52.5
**Unsatisfying economic status**	1808	59.8	61.0
**Housing Tenure**			
**Owner**	1991	63.9	67.2
**Owned by children**	664	21.3	18.1
**Rented and others**	463	14.9	14.7
**Smoking**	971	31.1	31.1
**Alcohol Drinking**	843	27.0	27.5
**Chewing betel nut**	259	8.3	9.3
**Leisure-time physical activity**			
**None**	1369	44.0	47.5
≤2 times/week, <30 mins/time	85	2.7	3.0
≤2 times/week, ≥30 mins/time	88	2.8	3.2
≥3 times/week, <30 mins/time	428	13.7	12.8
≥3 times/week, ≥30 mins/time	1145	36.8	33.5
**Eating breakfast**	2919	93.9	93.3

**Health check in the past three years**	1232	39.5	38.5
**Self-rated health (excellent and good)**	1447	47.7	48.2
**Hospitalization in the past year**	338	10.8	10.4
**Visual impairment**	255	8.5	8.1
**Hearing loss**	125	4.2	3.6
**Hypertension**	660	21.2	19.9
**Diabetes**	244	7.8	7.5
**Heart disease**	291	9.3	8.1
**Stroke**	37	1.2	1.1
**Cancers**	26	0.8	0.8
**Bronchitis**	217	7.0	6.4
**Arthritis**	405	13.0	12.6
**Gastrointestinal disorders**	393	12.6	12.8
**Liver or gall bladder diseases**	135	4.3	4.4
**Cataract**	373	12.0	9.8
**Glaucoma**	42	1.4	1.3
**Kidney diseases**	141	4.5	4.4
**Gout**	196	6.3	6.1
**Spin spur**	178	5.7	5.7
**Hip fractures**	23	0.7	0.8
**Other area fractures**	56	1.8	1.7
**Recall test**[§]	3008	4.6 (2.3)	4.7 (2.4)
**Backward digit test (no errors)**	2180	72.2	70.6
**CES-D**[§] **scores**	3008	4.3 (4.9)	4.3(5.1)
**Living alone**	242	7.8	6.7
**Social participation**	1490	48.5	46.9
